# The synergistic effect of calcium on organic carbon sequestration to ferrihydrite

**DOI:** 10.1186/s12932-018-0049-4

**Published:** 2018-02-03

**Authors:** Tyler D. Sowers, Jason W. Stuckey, Donald L. Sparks

**Affiliations:** 1 Department of Plant and Soil Sciences and Delaware Environmental Institute, University of Delaware, 221 Academy Street, ISE Lab, Newark, DE 19711 USA; 2 Biology Department, Multnomah University, 8435 NE Glisan St, Portland, OR 97220 USA

**Keywords:** Organic carbon, Iron oxide, Climate change, Ternary complexes, Soil

## Abstract

Sequestration of organic carbon (OC) in environmental systems is critical to mitigating climate change. Organo-mineral associations, especially those with iron (Fe) oxides, drive the chemistry of OC sequestration and stability in soils. Short-range-ordered Fe oxides, such as ferrihydrite, demonstrate a high affinity for OC in binary systems. Calcium commonly co-associates with OC and Fe oxides in soils, though the bonding mechanism (e.g., cation bridging) and implications of the co-association for OC sequestration remain unresolved. We explored the effect of calcium (Ca^2+^) on the sorption of dissolved OC to 2-line ferrihydrite. Sorption experiments were conducted between leaf litter-extractable OC and ferrihydrite at pH 4 to 9 with different initial C/Fe molar ratios and Ca^2+^ concentrations. The extent of OC sorption to ferrihydrite in the presence of Ca^2+^ increased across all tested pH values, especially at pH ≥ 7. Sorbed OC concentration at pH 9 increased from 8.72 ± 0.16 to 13.3 ± 0.20 mmol OC g^−1^ ferrihydrite between treatments of no added Ca^2+^ and 30 mM Ca^2+^ addition. Batch experiments were paired with spectroscopic studies to probe the speciation of sorbed OC and elucidate the sorption mechanism. ATR-FTIR spectroscopy analysis revealed that carboxylic functional moieties were the primary sorbed OC species that were preferentially bound to ferrihydrite and suggested an increase in Fe-carboxylate ligand exchange in the presence of Ca at pH 9. Results from batch to spectroscopic experiments provide significant evidence for the enhancement of dissolved OC sequestration to 2-line ferrihydrite and suggest the formation of Fe–Ca-OC ternary complexes. Findings of this research will inform modeling of environmental C cycling and have the potential to influence strategies for managing land to minimize OM stabilization.

**Electronic supplementary material:**

The online version of this article (10.1186/s12932-018-0049-4) contains supplementary material, which is available to authorized users.

## Background

On a global scale, soil organic matter (OM) sequesters more carbon (C) than vegetation and the atmosphere combined [1, 2]. Soil OM acts a reservoir for organic carbon (OC), making OM a vital component to ensuring soil health and productivity [1–4]. Instability of OM may lead to increased atmospheric C inputs, a primary mechanism of climate change [1, 2, 5, 6]. Historically, the stability of OM in soils and sediments was thought to be controlled largely by the chemistry of the OM present [2, 7]. However, this focal point of soil C cycling research has shifted over the past 15 years [8–10]. Recent research investigating soil C cycling has concentrated on how environmental processes such as organo-mineral interactions, soil physical properties, and microorganisms control the cycling of C in the environment [2, 11–13]. Metal oxides, found ubiquitously in soils, have been examined extensively due to a high reactivity and sorption affinity for soil OC [10, 13–16], displaying a high potential to drive the chemistry governing soil C cycling.

Metal oxides immobilize OC in soils by forming protective sorption complexes with OM [13, 15, 17–21]. Iron, Mn, and Al oxide minerals are primary OM-stabilizing soil constituents [13, 16, 18, 20]; however, Fe(III) oxide minerals are of particular environmental importance. Compared to Mn and Al oxides, Fe(III) oxides may sequester OC to a higher extent and may provide increased stability of sorbed OC [18, 22–26]. Although phyllosilicates also sequester OC, the degree of sorption is lower than that of Fe(III) oxide minerals by approximately an order of magnitude [27]. Poorly-ordered Fe(III) mineral phases, such as 2-line ferrihydrite, are of primary importance as these phases have the highest surface area and reactivity compared to more crystalline phases [9, 15, 23, 28, 29]. Also, sorption of dissolved OC to 2-line ferrihydrite has been found to be resistant to desorption under similar conditions to the initial OC sorption [15, 17]. The binding mechanism facilitating OC sorption to Fe(III) oxides has long been investigated using a variety of spectroscopic methods including attenuated total reflectance-Fourier transform infrared spectroscopy (ATR-FTIR), diffuse reflectance infrared Fourier transform spectroscopy (DRIFT), X-ray photoelectron spectroscopy (XPS), and X-ray absorption spectroscopy (XAS) [30]. Many of these studies suggest the sorption of OM to Fe(III) metal oxides occurs through a ligand exchange mechanism with the potential for additional outer-sphere complexation varying based on solution pH and ionic strength [15, 20, 31]. Ligand exchange occurs primarily through exchange of OC carboxyl functional moieties with hydroxyl groups [8, 13–15, 20, 32]. Aromatic groups are associated with Fe, though potentially through carboxyl attachment to aromatic rings [14]. However, the mechanism of OC sorption to metal oxides is still an area of intense research due to wide-ranging results concerning the reactivity of soil sorbents arising from the complexity of OM and technical difficulty in analyzing mechanisms dictating OC sequestration. Therefore, there is a need for further exploration of the organo-mineral associations controlling environmental C cycling. An important step forward in improving the current soil C cycling knowledge base is to investigate the cycling of OC in chemically complex ternary systems.

Much of the current understanding of OM sequestration in environmental systems has focused solely on binary systems of OC and Fe [13, 14]; however, the prevalence of divalent cations such as calcium (Ca) and magnesium (Mg) may affect the environmental cycling of C via ternary complex formation. The formation of bridging complexes between metal oxides and OM has been briefly discussed in the literature [20] but significant evidence of the occurrence and sorption behavior of these complexes in environmental systems is scare. Recent research on ternary complex formation in systems containing iron oxides have been found to play a role in arsenate and phosphate sequestration. Antelo et al. [33] found increasing Ca concentration in ferrihydrite and arsenate/phosphate systems resulted in increased arsenate/phosphate sequestration at pH ≥ 8 along with a corresponding decrease in aqueous Ca concentration. The results observed in this study provide a promising analogue to research probing the sequestration of OC as Fe–Ca-OM ternary complexes. Weng et al. [10], for example, suggests that OC sorption to metal oxides may exhibit behavior similar to polyvalent ions such as phosphate, which implies that potential ternary complexation behavior for ferrihydrite, Ca, and OC may occur similarly to reported ferrihydrite, Ca, and phosphate ternary complexes [33]. Preliminary evidence exists for the presence of Ca associations with Fe and OC in environmental systems. The use of synchrotron-based scanning transmission X-ray microscopy (STXM) has shown significant correlation between Ca and C in the clay fraction of soils that contain high Fe content but do not have carbonate minerals [8]. Humic acid sorption to Fe(III) minerals is promoted by the presence of Ca [34, 35]. These results in conjunction with the known association of Fe and OM suggest that Ca may be an important factor determining OC sequestration and deserve further investigation.

Research probing the mechanism and sequestration extent of OC sequestration to Fe oxide is needed to reliably model C cycling in environmental systems. Examining the effect of polyvalent ions such as Ca on OC sequestration could have major impacts on how C cycling is modeled in natural systems. To explore the effect of Ca on OC sorption to Fe oxides, we 1) determined the impact of Ca on dissolved organic carbon (DOC) sorption on 2-line ferrihydrite and 2) probed the mechanism of expected Fe–Ca-OC ternary complexes. We expected that Ca enhanced the sorption extent of OC through the formation of bridging structures between Fe and OC. The following research strives to provide chemical evidence for the potential formation of Fe–Ca-OC ternary complexation.

### Natural and laboratory sources of organic carbon

Both natural and model (citric acid) organic carbon sources were tested to determine the effect of Ca on OC sorption to synthetic 2-line ferrihydrite. Water-extractable dissolved organic matter (DOM) was used as a natural source of OC. Leaf litter used for the DOM extraction was obtained from the Stroud Water Research Center (Avondale, PA). Samples were collected from the top 10 cm of leaf litter from a forest soil O_a_ horizon (Typic Hapludult). Using a method adapted from Chen et al. and Stuckey et al. [14, 26], soil-organic materials were mixed with DI water for 90 h (1:2 fresh leaf litter:DI water [w/w]) while being vigorously stirred (200 rpm) on a rotary shaker. After a 90-h equilibration time, the resulting solution was centrifuged (20,000*g* for 1 h) and sequentially vacuum filtered through polyethersulfone filters of 0.8, 0.45, and 0.2 µm pore size. Sequential filtration was performed due to the large range in particle size of the leaf litter extract. The filtered DI-extractable DOM solution was then analyzed using a total organic carbon (TOC) analyzer (Apollo 9000 series) to determine OC concentration (Additional file 1: Table S1). A metal/metalloid elemental analysis of the DOM solution was performed using inductively coupled plasma-atomic emission (ICP-AES) (Additional file 1: Table S2). Citric acid was chosen as a model OC compound analogue for our study. Citric acid (Sigma Aldrich) is a tricarboxylic acid and was chosen due to the high reactivity of the carboxylic acid moiety, high concentration of carboxylic acid from DOM extracted from the same leaf litter site [14], and the favorable sorption of carboxylic acid to iron oxides [13, 15]. Attenuated total reflectance-Fourier transform infrared spectroscopy (ATR-FTIR) analysis of the DOM (Table 2) confirmed that carboxylic acid is the dominant C moiety present (assignment), with aromatic (1585 cm^−1^), phenolic (1270 cm^−1^), and polysaccharide (1120, 1080, and 1040 cm^−1^) groups present to a lesser degree.

### Synthetic 2-line ferrihydrite synthesis

Two-line ferrihydrite, a ubiquitously found Fe(III) oxide, was synthesized according to procedures established in Cornell and Schwertmann [23]. 40 g of Fe(NO_3_)_3_∙9 H_2_O was dissolved in 500 mL of deionized (DI) water. In order to bring the pH to a range of 7–8, 330 mL of 1 M KOH was added, while stirring, to a Fe(NO_3_)_3_∙9 H_2_O solution. The pH of the solution was continuously monitored during the addition of the final 20 mL of 1 M KOH. Upon reaching this pH range, the solution was centrifuged and dialyzed, yielding 10 g of 2-line ferrihydrite.

### Sorption isotherms

Multiple adsorption isotherm experiments were performed to test the effect of increasing DOC concentration and/or the effect of increasing Ca concentration on OC sorption to synthetic 2-line ferrihydrite. The previously discussed DOM stock was used as a source of OC for all adsorption experiments. Prior to beginning sorption experiments, the DOM stock was determined to have an OC concentration of approximately 2000 mg OC L^−1^ and a Ca concentration of approximately 4 mM Ca. Each reactor received 43 mg of 2-line ferrihydrite as a wet paste and was suspended with 40 mL of DOM stock diluted with Type 1 deionized (DI) water to achieve a series of initial OC concentrations, such that the C/Fe molar ratio ranged from 0.3–16.9. Initial Ca concentration increased as initial DOM concentration increased due to the inherent Ca concentration present in the DOM stock, resulting in Ca concentration increasing up to 4 mM Ca for the most concentrated DOM sample (initial C/Fe molar ratio of 16.9). The pH of the suspensions was adjusted to 6.25 ± 0.10 using NaOH and/or HCl in order to perform experiments at an environmentally relevant pH. All samples were covered and mixed via a rotary shaker (50 rpm) in the dark for 24 h after pH adjustment. The 24-h equilibration time was determined by performing preliminary sorption experiments, in which maximum OC sorption was reached within 24 h. Sample pH was monitored intermittently throughout the equilibration time to ensure that the pH remained at 6.25 ± 0.10. Upon completion of the 24-h equilibration, samples were centrifuged (20,000*g*) and the supernatant was collected for TOC and ICP-AES analysis. Solid samples were washed at least twice with DI water and stored moist in a freezer at approximately − 4 °C until further analysis. Analogous control experiments were performed with citric acid as the C source rather than natural DOM. All sorption isotherm experiments here and throughout were performed in at least duplicate.

For the next series of sorption isotherm experiments, the previously detailed procedure was repeated, expect CaCl_2_ was added to each reactor such that the final Ca concentration was equal to the Ca concentration of the sample receiving the greatest concentration of DOM solution (~ 4 mM Ca). For citric acid experiments, which contain no native Ca concentration, CaCl_2_ was added to match the Ca concentration of the DOM experiments. Lastly, separate sorption isotherms testing the effect of increasing Ca concentrations were performed for C/Fe molar ratios of 4.7 and 12.5. Calcium chloride was added to each sample such that the initial Ca concentration ranged from approximately 1 mM Ca to 60 mM Ca. All other steps were carried out as described for the 4 mM Ca sorption experiments. Prior to performing experiments with increasing Ca concentration, a subsample of stock DOM solution was equilibrated with 100 mM Ca (via CaCl_2_) to ensure no precipitates formed. No precipitates were formed after shaking for a 24-h equilibration time.

TOC analysis was performed before and after sorption of DOM to 2-line ferrihydrite for all experiments. The quantity of OC sorbed was calculated by measuring the difference between the OC concentrations before and after sorption. Sorption isotherm data were processed and fit to the Langmuir equation using a preprogrammed Excel sheet [36].

### Sorption envelopes

The effect of pH and Ca concentration at an initial C/Fe molar ratio of 4.7 was determined by conducting sorption envelope experiments with DOM and 2-line ferrihydrite. The experimental setup was equivalent to what was performed for sorption isotherm reactions discussed previously; however, initial OC concentration was consistent across all samples and pH was the variable being changed. All samples contained 43 mg of 2-line ferrihydrite and were suspended in the DOM stock solution such that the initial C/Fe molar ratio equaled 4.7. Subsequently, sample pH was adjusted with HCl and NaOH (accounted for < 1% of total solution volume) from pH 4 to 9. All envelope experiments were then shaken in the dark for 24 h and then sampled according to the sorption isotherm procedure discussed earlier. Separate envelope experiments were performed at different Ca concentrations (via CaCl_2_ addition), which consisted of approximately 1 (background DOM concentration), 10, or 30 mM Ca in order to capture typical behavior at high soil porewater Ca concentrations [33, 37]. Control sorption envelope experiments were performed with only Ca and 2-line ferrihydrite to evaluate Ca sorption to ferrihydrite without DOM. Aqueous and solid samples from the sorption envelope experiments were collected after 24 h of reaction time and analyzed similarly to what was described earlier for sorption isotherm experiments. Before beginning envelope experiments, subsamples of DOM stock were adjusted from pH 4 to 9 to determine if any precipitates formed. No precipitates were observed after 24 h.

### ATR-FTIR spectroscopy

Freeze-dried DOM and ferrihydrite samples were analyzed using a Bruker ATR-FTIR. Spectra were scanned from 4000 to 600 cm^−1^ at a spectra resolution of 2 cm^−1^. 2-line ferrihydrite spectra was subtracted from all OC-bearing ferrihydrite samples to remove contributions from ferrihydrite to focus on the chemical composition of the bound OC. Automatic baseline correction and normalization was applied to all spectra. All samples were dried and analyzed soon after to avoid the effects of moisture uptake on sample spectra. The OPUS Version 7.2 spectroscopy software suite (Bruker) was used to process all collected spectra.

### Additional characterization techniques

X-ray diffraction (XRD) and Brunauer–Emmett–Teller (BET) analysis were performed to confirm the mineralogy and external surface area of synthesized 2-line ferrihydrite, respectively. A Bruker D8 Discover diffractometer was used to perform XRD analysis. A synthesized iron oxide sample was freeze-dried prior to analysis and analyzed from 20° to 75° 2θ (step size 0.05° 2θ). The analysis confirmed the synthesis of 2-line ferrihydrite (Additional file 1: Figure S1). The BET surface area of synthesized 2-line ferrihydrite was found to be 288.57 m^2^ g^−1^, which is within the BET surface area range reported for synthetic ferrihydrite [23]. A vario Micro cube CHNS analyzer was used for a subset of OC-bearing ferrihydrite samples to confirm that the quantity of sorbed C in the solid phase was consistent with that removed from the liquid phase before and after reaction (data not shown).

A subset of sorption samples was digested to supplement aqueous Fe and Ca data. A 10 mg sample of 2-line ferrihydrite was digested using aqua regia (3:1 molar ratio of HCl and HNO_3_) and measured for Fe and Ca using ICP-AES.

## Results and discussion

### Effect of Ca on the sorption extent of DOM and citric acid to ferrihydrite

**Fig. 1:**
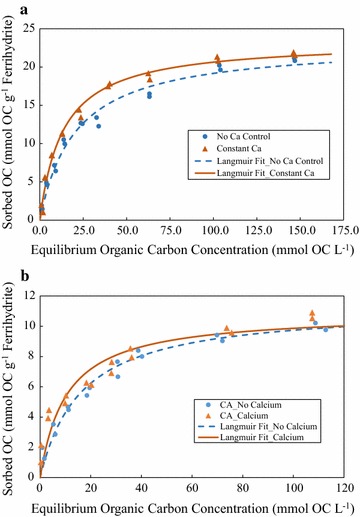
Sorption behavior of organic carbon to 2-line ferrihydrite: DOM (**a**) and citric acid (**b**) were reacted with ferrihydrite at pH 6.25 ± 0.10 in duplicate. Samples were treated with and without Ca addition. Sample receiving Ca had a total Ca concentration of 4 mM Ca (152 mg Ca L^−1^)

All isotherm data was well described by the Langmuir equation with OC sorbed concentrations increasing rapidly at low solution OC concentrations, with decreasing slope at high equilibrium concentrations (Fig. 1, Table 1). This sorption phenomenon is consistent with the L-type isotherm; therefore, the Langmuir equation was chosen over the Freundlich to fit all sorption isotherm data as the Langmuir equation was found to have a superior goodness of fit [5, 38]. All Langmuir fitting parameters ($S = (S_{{\text{max}}} KC)/\left( {1 + KC} \right)$ where S is the sorbed concentration, S_max_ is the predicted maximum sorbed concentration, $K$ is a sorption constant, and $C$ is the equilibrium sorbate concentration) are shown in Table 1.

**Table 1:** Langmuir parameters used to model sorption of DOM and citric acid to 2-line ferrihydrite

Organic C source	Calcium treatment	S_max_ (mmol OC g^−1^ ferrihydrite)	K	E
DOM	No Ca control	23.13 ± 0.7455	0.004 ± 0.001	0.978
	4 mM Ca	23.41 ± 0.5125	0.006 ± 0.001	0.989
Citric acid	No calcium	11.32 ± 0.4690	0.005 ± 0.001	0.967
	4 mM Ca	10.89 ± 0.6903	0.008 ± 0.002	0.906

*S*_*max*_ predicted maximum sorbed concentration; *K* sorption constant; *E* model efficiency

In Fig. 1a, DOM sorption to 2-line ferrihydrite data was fit using the Langmuir equation with a high model efficiency for the data sets with and without Ca control (0.978 and 0.989, respectively). The Langmuir parameters for the non-Ca controlled and Ca controlled data sets were similar. Two differing sets of isotherms, one where DOM was reacted with 2-line ferrihydrite without the addition of CaCl_2_ and the other where CaCl_2_ was added such that Ca concentration was constant across all initial C/Fe concentrations, were performed to determine the effect of Ca control on DOM sorption on 2-line ferrihydrite. The maximum sorbed OC concentration for both data sets was observed to be 23.13 mM OC for the most concentrated DOM sample (initial C/Fe molar ratio of 16.9). The observed sorption maximum is mostly consistent with what Chen et al. observed at pH 7, although our experiments resulted in approximately 10% higher sorption relative to a system without exogenous calcium [14]. Although the Langmuir fitting parameters for the data sets were similar, the sorbed OC concentration from 25 to 75 mM equilibrium OC for the Ca controlled isotherm was found to differ by approximately 10% to 15%. The difference in sorption at the 25 to 75 mM equilibrium OC concentration range was attributed to differences in Ca concentration (~ 1 mM Ca for non-Ca controlled isotherm samples versus ~ 4 mM Ca for Ca-controlled isotherm samples) (Fig. 1).

Analogous sorption isotherm experiments, with and without fixed Ca concentration, were conducted using citric acid as an OC source. The sorption extent of citric acid to 2-line ferrihydrite was significantly less than that of DOM (S_max_ = 11.32). The decreased affinity of citric acid to iron oxides compared to natural DOM is supported by the literature, although the observed sorption maximum for citric acid experiments was approximately an order of magnitude greater than that of citric acid sorption to goethite experiments [39]. The lesser reactivity and surface area of goethite compared to 2-line ferrihydrite is the most likely reason for the observed disparity in sorption maximum. Unlike what was observed for DOM experiments, no significant differences in sorption extent were observed between data sets of no Ca addition and 4 mM Ca addition. These results suggest that Ca does not have an effect on the sorption of OC as citric acid to 2-line ferrihydrite. The DOM source has been found to consist of predominately carboxylic functional moieties through ATR-FTIR (Fig. 5) and past studies [14]; therefore, it is surprising that citric acid sorption behavior differs from DOM. Chemical heterogeneity of natural DOM compared to a simple model compound such as citric acid is a probable reason for the observed difference and suggests that citric acid may not be a representative analogue when modeling OC cycling in natural systems.

### Effect of pH and Ca concentration on DOM sorption

**Fig. 2:**
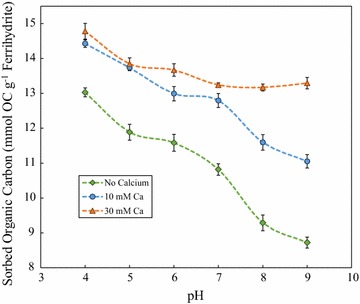
The effect of pH on DOM sorption to 2-line ferrihydrite: sorption envelopes of DOM sorption to ferrihydrite at pH 4 to 9 in the presence of no added Ca (**a**), 10 mM Ca (**b**), or 30 mM Ca (**c**). An initial C/Fe ratio of 4.7 was used for all samples and experiments were performed in triplicate

Sorption envelope experiments were performed to determine the concentration of sorbed OC, as DOM, to 2-line ferrihydrite from pH 4–9 at differing initial Ca concentrations (Fig. 2). An initial C/Fe molar ratio of 4.7 was used for all sorption envelope experiments and zero Ca (native concentration of ~ 1 mM Ca from DOM), 10 mM Ca, or 30 mM Ca were added. The sorption data for the zero added Ca treatment decreased from a maximum of 13.03 ± 0.12 mmol OC g^−1^ 2-line ferrihydrite as pH increased, with a major decrease near the 2-line ferrihydrite point of zero charge (PZC = ~ 7.5). The lowest sorbed OC concentration was found to be 8.72 ± 0.16 mmol OC g^−1^ 2-line ferrihydrite at pH 9. The decrease in sorbed OC concentration is best explained by the effects of pH on the variable charge of 2-line ferrihydrite. The 2-line ferrihydrite mineral surface should become less positively charged with increasing pH until the PZC is reached, after which the mineral surface becomes increasingly negatively charged [5, 23, 26, 38]. However, the addition of Ca to the system resulted in major differences in sorbed OC concentration, especially at pH > PZC.

The addition of ≥ 10 mM Ca simultaneously with DOM to the 2-line ferrihydrite system resulted in increased sorbed OC concentration across all tested pHs. The 10 mM Ca treatment resulted in approximately 2 mmol OC g^−1^ 2-line ferrihydrite more sorption from pH 4 through 7 compared to the data set with no Ca treatment. The 30 mM Ca treatment resulted in similar sorbed OC concentration compared to the 10 mM Ca treatment from pH 4 through 7, with approximately 0.5 mmol OC g^−1^ 2-line ferrihydrite increased sorption at pH 6 and 7. At pH ≥ 7 however, all sorbed OC concentration data significantly differed between data sets. Sorbed OC concentration differed greatest between sets at pH 9, with observed OC sorbed concentrations of 8.72 ± 0.16, 11.05 ± 0.19, and 13.3 ± 0.20 mmol OC g^−1^ 2-line ferrihydrite for the no Ca, 10 mM Ca, and 30 mM Ca treatments, respectively. Another important feature in Fig. 2 is that the 30 mM Ca data set was found to vary only slightly across the pH 5–9 range. Sorbed OC concentration of samples at pH 4 and 5 varied by approximately 0.6 ± 0.2 mmol OC g^−1^ 2-line ferrihydrite, whereas samples at pH 7–9 varied by only approximately 0.15 ± 0.044 mmol OC g^−1^ 2-line ferrihydrite.

The higher sorption of OC with Ca addition and the lower variation in sorbed OC from pH 5–9 for the 30 mM Ca data set suggests that Ca enhances sequestration of OC to 2-line ferrihydrite. Trends similar to those observed in Fig. 2 were observed in systems studying the effect of initial Ca concentration on phosphate/arsenate sorption to 2-line ferrihydrite [33]. Antelo et al. observed high disparity in phosphate sorption concentration at pH ≥ 7 with increasing Ca concentration, the same trend observed in our study for OC sorption concentration. Also, the rate of phosphate sorption decrease declined dramatically with increasing pH at 6 mM Ca; therefore, the effect of Ca on phosphate sorption to ferrihydrite appears to be similar to the ferrihydrite-Ca-OC system. The similarity in behavior between phosphate and OM sorption to Fe oxide has been shown in prior research as well [10], suggesting that ferrihydrite-Ca-phosphate systems may be an appropriate analogue when examining ferrihydrite-Ca-OC systems. The most likely explanation for the observed synergistic effect of Ca on OC sorption is due to the potential formation of Ca-bridging between ferrihydrite and OC. The high concentration of a divalent cation such as Ca^2+^ in a system containing negatively charged DOM and ferrihydrite surfaces at pH ≥ 7 may facilitate the bridging process and would explain the consistent OC sorption concentration from pH 5–9 of the 30 mM Ca data set. It was unexpected that OC sorption concentration would be affected by Ca concentration at pH 4 and 5 due to the system being electrostatically unfavorable for the formation of outer sphere complexes. Ligand exchange processes may be controlling the Ca effect on OC sorption.

### Coupled Ca concentration decrease with increasing pH in batch reactor systems

**Fig. 3:**
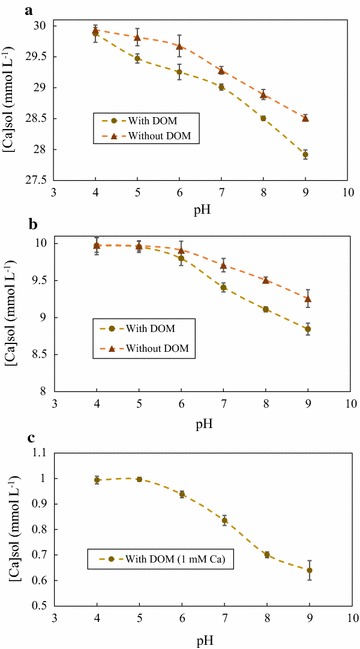
Calcium sorption behavior on 2-line ferrihydrite: aqueous Ca data from sorption envelope experiments at initial Ca concentrations of 30, 10, and 1 mM Ca (**a**, **b**, and **c**, respectively) conducted from pH 4 to 9. The multicomponent systems consisted of Ca and ferrihydrite or Ca, ferrihydrite, and DOM. The greater decrease in aqueous Ca in the DOM multicomponent system compared to the system without DOM is thought to occur due to ternary interactions of ferrihydrite, Ca, and DOM

Initial Ca concentrations of 1 mM Ca (baseline Ca concentration from DOM), 10 mM Ca, and 30 mM Ca were tested in 2-line ferrihydrite systems where DOM was added simultaneously with Ca (Fig. 3). Calcium concentration remained insignificantly changed in the absence of DOM at pH 4–6 for Fig. 3a, b. Above pH 6, aqueous Ca concentration decreased until aqueous Ca concentrations of approximately 28.5 mM Ca and 9.25 mM Ca (for Fig. 3a, b, respectively) at pH 9. Antelo et al. also observed no significant change in aqueous Ca concentration in systems equilibrated with 2-line ferrihydrite at acidic pH and a decrease in aqueous Ca concentration as pH increased [33]. However, Anetelo et al. determined a significant decreases in aqueous Ca concentration began at approximately pH 8.5, which deviates from our observation of aqueous Ca concentration decreasing beginning at approximately pH 7. The disparity is most likely due to differences in experimental setup and potentially differences in mineral charge. The PZC of 2-line ferrihydrite supports our observations as the 2-line ferrihydrite should become negative at pH higher than approximately 7–7.5. In the presence of DOM, a similar trend was observed for Fig. 3a, b; however, aqueous Ca concentration was lower for a majority of the tested pH range. In Fig. 3a, aqueous Ca concentration was similar at pH 4 for the data sets with and without DOM but the data set with DOM began to decrease to a more significant extent (up to ~ 0.6 mM Ca difference) than the data set without DOM. A similar trend was observed for Fig. 3b; however, the difference between data sets was within standard error from pH 4–6. Differences in aqueous Ca concentration between the data sets with and without DOM increased up to 0.45 mM Ca at pH 9. Aqueous Ca concentration data in Fig. 3c was performed at the baseline Ca concentration (~ 1 mM Ca) that was present in all sorption experiments (with no added Ca) at an initial C/Fe molar ratio of 4.7. Aqueous Ca concentration decreased at the greatest rate from pH 6–8 and at pH 9, reaching an aqueous Ca concentration minimum of 0.64 mM Ca.

Aqueous Ca data from Fig. 3 correlate trends observed in Fig. 2. Aqueous Ca concentration decreased to a greater extent than the Ca-ferrihydrite control for Fig. 3a, b, which coincides with increased sorption of DOM at high pH and increasing Ca concentration observed in Fig. 2. Decrease in aqueous Ca concentration is expected with increasing pH due to the 2-line ferrihydrite mineral surface becoming increasingly positively charged. However, the observed lower aqueous Ca concentration in the presence of DOM suggests that Ca may be interacting with both the DOM and 2-line ferrihydrite.

### Increased DOM sequestration with Increasing Ca concentration

**Fig. 4:**
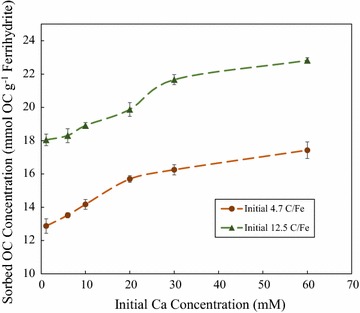
The effect of calcium concentration on DOM sorption to 2-line ferrihydrite: sorption of DOM to 2-line ferrihydrite at 4.7 and 12.5 initial C/Fe molar ratios with increasing calcium concentration (up to 60 mM Ca) at pH 6.25 ± 0.10

The effect of initial Ca concentration was further explored by performing separate DOM sorption experiments for initial C/Fe molar ratios of 4.7 and 12.5 with increasing Ca concentration (Fig. 4). Both the 4.7 and 12.5 C/Fe data sets were observed to have similar trends in sorbed OC concentration with initial Ca concentration ranging up to 60 mM Ca. Sorbed OC concentration increased linearly with increasing Ca concentration until an initial Ca concentration of 20 mM and then began to plateau at approximately 4.5 mmol OC g^−1^ 2-line ferrihydrite greater than samples receiving zero initial Ca. Initial Ca concentrations greater than 60 mM Ca are expected to continue to increase sorbed OC concentration but to a lesser extent than what is observed from 0 to 60 mM Ca. Figure 4 provides further evidence that Ca is enhancing the sequestration of DOM to 2-line ferrihydrite.

### ATR-FTIR spectroscopy

**Fig. 5:**
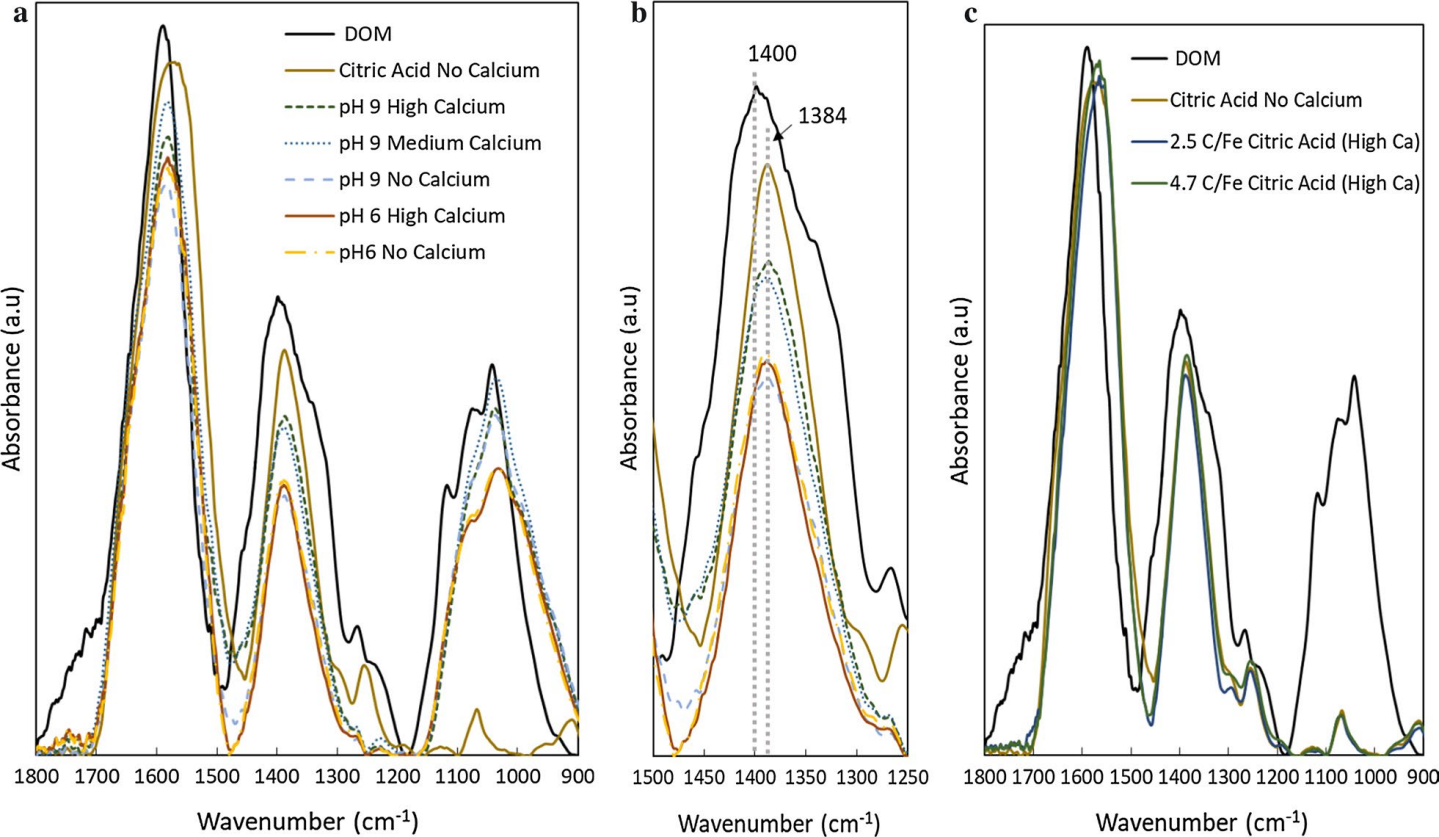
ATR-FTIR spectra for DOM-bearing 2-line ferrihydrite with and without calcium addition: background corrected and normalized FTIR spectra for DOM (**a** and **b**) or citric acid-bearing (**c**) 2-line ferrihydrite in the presence or absence of Ca at either pH 6 or 9. Samples with Ca at pH 9 were observed to have increased peak area and a broader peak compared to those without Ca, which indicates increased association of 2-line ferrihydrite with COO^−^ functional moieties

Distinct differences and similarities were observed for ATR-FTIR spectra of sorption complexes formed at differing initial chemical conditions (Fig. 5). Due to the increased sorption of OC at pH ≥ 7 with increasing Ca concentration (Fig. 2), OC-bearing ferrihydrite reacted at pH 9 was analyzed and compared to samples reacted at pH 6. Past work performing FTIR analysis on C sorption to Fe oxides has shown major effects of sorption on asymmetric and symmetric COO^−^ (Table 2), which was also observed in our system. The asymmetric COO^−^ band (Fig. 5a) for all sorption complexes shifted from 1585 cm^−1^ (band location for DOM spectra) to approximately 1570 cm^−1^. Calcium treatment and pH did not have an observed effect on the asymmetric band across all samples. However, revealing differences were seen in spectra with increased Ca and pH at the symmetric COO^−^ band. The DOM spectrum was found to have a symmetric band at 1400 cm^−1^, which is analogous to other FTIR studies with organic matter [39–41]. A distinct shift from 1400 to 1384 cm^−1^ (Fig. 5b) was observed for all sorption samples. Strong evidence exists among OC sorption studies that the shift of the symmetric COO^−^ band, in conjunction with a shift of the asymmetric band, to 1384 cm^−1^ is an indicator of an Fe to carboxylate inner sphere ligand exchange mechanism [14, 39, 40]. Unlike the asymmetric band, OC-bearing ferrihydrite samples formed at pH 9 in the presence of 10 or 30 mM Ca (medium and high, respectively) were found to have broader peaks with increased peak area compared to samples formed at pH 6, regardless of initial Ca concentration, and pH 9 with no Ca concentration. The increased, broader band at 1384 cm^−1^ for samples pH 9 Medium Calcium (10 mM Ca) and pH 9 High Calcium (30 mM Ca) signifies the increased occurrence of Fe-COO bond formation [39, 40, 42]. This spectral evidence suggests that Ca is promoting increased association of Fe and carboxylic functional moieties at pH 9, which may be occurring due to the formation of Fe–Ca-carboxylate ternary complexes.

**Table 2:** Observed peak positions for ATR-FTIR spectra. Peak assignments and references supporting assignment are included

Peak position (cm^−1^)	Peak assignment	References
1585–1570	Asymmetric COO^−^ stretch	Heckman et al. [42]; Lackovic et al. [39]
1530–1520	Aromatic C=C stretch	Chen et al. [8, 14]; Gu et al. [41]
1400	Symmetric COO-stretch	Gu et al. [41]; Fu and Quan [40]; Heckman et al [42]; Lackovic et al. [39]
1384	COO-Fe stretch	Chen et al. [8, 14]; Fu and Quan [40]; Lackovic et al. [39]
1270	Stretching of phenolic OH	Gu et al. [41]; Artz et al. [46]; Chen et al. [8, 14]
1250	OH deformation of COOH	Gu et al. [8, 14]; Heckman et al. [42]
1120	Polysaccharides: C–O stretch	Chen et al. [8, 14]; Heckman et al. [42]
1080	Polysaccharides: C–O stretch	Artz et al. [46]; Chen et al. [8, 14]; Heckman et al. [42]
1040	Polysaccharides: C–O stretch	Chorover and Amistadi [27]; Grube et al. [47]

Aromatic (1530–1520 cm^−1^) and phenolic (1270 cm^−1^) bands present in DOM spectra were severely diminished or not observed for OC-bearing ferrihydrite sample spectra, suggesting that these functional moieties were not significantly involved in the sorption of OC to ferrihydrite in our study. Bands at 1120, 1080, and 1040 cm^−1^ indicate the presence of polysaccharides in the DOM spectra. Other than the 1120 cm^−1^ band, the 1080 and 1040 cm^−1^ bands were observed in sorption sample spectra, but with dampened peak intensity. The presence of these peaks suggests that polysaccharide association with ferrihydrite is occurring; however, the association is weak given the low peak intensity and the lack of a shift in the band position. Further examination of these features revealed that all pH nine samples exhibited increased intensity compared to those formed at pH 6. The increased association of polysaccharides with ferrihydrite at pH 9 most probably is explained by favorable outer-sphere complexation between OC and ferrihydrite at high pH, due to the presence of Ca^2+^ (the pH 9 No Calcium sample has a baseline 1 mM Ca concentration from the DOM solution) and the negative surface charge of ferrihydrite (PZC of ~ 7–7.5) at pH 9 [5, 43].

Citric acid-bearing ferrihydrite was also analyzed (Fig. 5c). Shifts similar to what were observed for DOM-bearing ferrihydrite samples were found at the asymmetric and symmetric COO^−^ bands. The bands had a high peak intensity as well, suggesting strong binding to ferrihydrite. However, no significant differences in spectra features were observed for samples that received Ca, suggesting that the addition of Ca has little effect on the binding of citric acid to ferrihydrite.

### Potential formation of Fe–Ca-DOM ternary complexes

The previously discussed batch reactor and ATR-FTIR experiments provide evidence for the synergistic effect of Ca on DOM sequestration to 2-line ferrihydrite. However, the question remains as to what processes are controlling the increase in DOM sorption in the presence of Ca. Antelo et al. through a combination of batch reaction and modeling studies with ferrihydrite, Ca, and arsenate/phosphate, attributed the synergistic effect of Ca on phosphate sorption to Fe–Ca-phosphate ternary complex formation [33]. Organic carbon sorption envelope data paired with corresponding aqueous Ca data (Figs. 2, 3, respectively) were observed to have similar trends that were observed for phosphate in Anetelo et al. Results from both systems show that Ca addition limits the decrease in OC, or phosphate, sorption with increasing pH. The congruent results of both systems suggest that our system may also facilitate the occurrence of ternary complexes. Also, the formation of Ca-bridging at high pH has been thought to potentially have an effect on OM sorption to iron oxides due to the decrease in positively charged surface sites, and subsequent increase in negatively charged surface sites at pH ≥ 7 [23, 38]. Significant concentrations of a divalent cation such as Ca^2+^ in the presence of negatively charged OM and a negatively charged ferrihydrite surface may facilitate the bridging processes due to electrostatic favorability [5, 30]. In addition to potential ternary complex formation, it is also possible that OC coatings onto ferrihydrite are being formed in the presence of Ca. Due to the high concentration of OC and increased Ca adsorption in the presence of DOM, moieties of OC may be bridged by Ca which in turn could promote the formation of OC coatings.

In our study, ATR-FTIR spectroscopy spectra support the ternary associations of Fe, Ca, and OC. Calcium addition at pH 9 increased symmetric COO^−^ peak intensity compared to samples reacted at pH 6 or pH 9 with no Ca addition, suggesting greater association of carboxylic moieties of the DOM with ferrihydrite samples reacted with Ca at pH 9. Also, the shift of the symmetric COO^−^ band to 1384 cm^−1^ provides evidence that a ligand exchange mechanism is occurring between Fe and COO^−^. This shift occurs for all samples, but the distortion of the Ca-receiving spectra suggests the increased occurrence of Fe-COO bond formation. Outer-sphere complexes are expected as well due to the electrostatics of the system, especially at high pH. Polysaccharide sorption to ferrihydrite may account for a portion of the outer sphere complexes, if it is occurring, due to increased peak area with no shift at 1080 and 1040 cm^−1^ at pH 9 compared to pH 6. However, further spectral analysis is needed to pair with the evidence found using ATR-FTIR to continue to probe the mechanism(s) controlling the increased sequestration of OC in the presence of Ca.

## Conclusions

Our results suggest that Ca enhances the extent of OC sorption to 2-line ferrihydrite from pH 4–9, especially at pH ≥ 7. Results from all batch experiments provides evidence for the increased sequestration of OC up to a Ca concentration of 60 mM Ca (Fig. 4). This result aligns with our initial hypothesis as we predicted the increased sequestration of OC with Ca amendment due to the proposed formation of Fe–Ca-OC ternary complexes. Citric acid sorption to 2-line ferrihydrite was not significantly affected by the presence of Ca. ATR-FTIR spectroscopic analysis provides evidence for the occurrence of ligand exchange of carboxylate functional moieties to the 2-line ferrihydrite surface and also suggests that Ca may be promoting the increased formation of Fe-COO bonds. Outer-sphere complexation is most likely involved as well but is unable to be directly proven from the ATR-FTIR spectra. It is also possible that Fe-OC-Ca may form at acidic pH due to the observed increased Ca adsorption at low pH in the presence of DOM and the expected positive charge of ferrihydrite below the PZC; however, further batch and spectroscopic work would need to be performed to prove the relative contribution of each potential ternary complex.

The proposed synergistic effect of Ca on DOM sequestration to 2-line ferrihydrite has major implications on how C cycling should be modeled in terrestrial systems. Understanding the role of Ca in systems containing both DOM and Fe oxides may provide a key component needed to accurately model the C cycle. Specifically, this work may have significant implications on C sequestration in soils. The common use of Ca-containing soil amendments may have a beneficial effect on OC retention in soils with significant concentrations of Fe oxide, which in turn may affect soil fertility. Addition of Ca^2+^ via lime application has been observed to increase OM content in field studies and was attributed to the high concentration of polyvalent cations facilitating decrease of the diffuse double layer, ultimately resulting in increased aggregation [44, 45]. Our work may provide a chemical explanation for increased OC sequestration in Ca-amended soil systems in conjunction with the already proposed physical protection via aggregation. This work is potentially applicable to forest soils as well, due to the source of the natural DOM used in this study, and may prove useful when conducting C research in forest environments. Future work to be performed on this project will focus on analyzing ferrihydrite-Ca-DOM samples by conducting desorption experiments to determine the stability of generated complexes and using a variety of advanced X-ray absorption spectroscopic techniques to provide further evidence of the mechanism of DOM sorption in ternary systems.

## Additional file

**Additional file 1: Figure S1.** XRD data for synthesized 2-line ferrihydrite. The two labeled broad peaks are characteristic of 2-line ferrihydrite and confirmed we were using the correct Fe mineral phase. **Table S1.** Aqueous carbon concentrations of DOM stock solution. **Table S2.** Aqueous elemental composition of DOM stock solution.

## Abbreviations

C: carbon
OC: organic carbon
DOC: dissolved organic carbon
DOM: dissolved organic matter
Fe: iron
Ca: calcium
ATR-FTIR: attenuated total reflectance-Fourier transform infrared spectroscopy
COO^−^: carboxylate anion of a carboxylic acid
DRIFT: diffuse reflectance infrared Fourier transform spectroscopy
XPS: X-ray photoelectron spectroscopy
XAS: X-ray absorption spectroscopy
STXM: scanning transmission X-ray microscopy
XRD: X-ray diffraction
