# Supplementary material for: The synergistic effect of calcium on organic carbon sequestration to ferrihydrite
Source: Geochem Trans. 2018 Feb 3;19:4. doi: 10.1186/s12932-018-0049-4 (PMC5797485; doi:10.1186/s12932-018-0049-4)
Supplement: Supplementary file 1 — Additional file 1: Figure S1. XRD data for synthesized 2-line ferrihydrite. The two labeled broad peaks are characteristic of 2-line ferrihydrite and confirmed we were using the correct Fe mineral phase. Table S1. Aqueous carbon concentrations of DOM stock solution. Table S2. Aqueous elemental composition of DOM stock solution. [file 12932_2018_49_MOESM1_ESM.docx]

Additional Information

The Synergistic Effect of Calcium on Organic Carbon Sequestration to Ferrihydrite

Tyler D. Sowers^a*^, Jason W. Stuckey^ab^, & Donald L. Sparks^a^

Department of Plant and Soil Sciences and Delaware Environmental Institute, University of Delaware, 221 Academy Street, ISE Lab, Newark, Delaware 19711, USA^a^

Multnomah University, 8435 NE Glisan St, Portland, OR 97220, USA^b^

[tdsowers@udel.edu](mailto:tdsowers@udel.edu) (Corresponding author*)

[jstuckey@multnomah.edu](mailto:jstuckey@multnomah.edu)

[dlsparks@udel.edu](mailto:dlsparks@udel.edu)


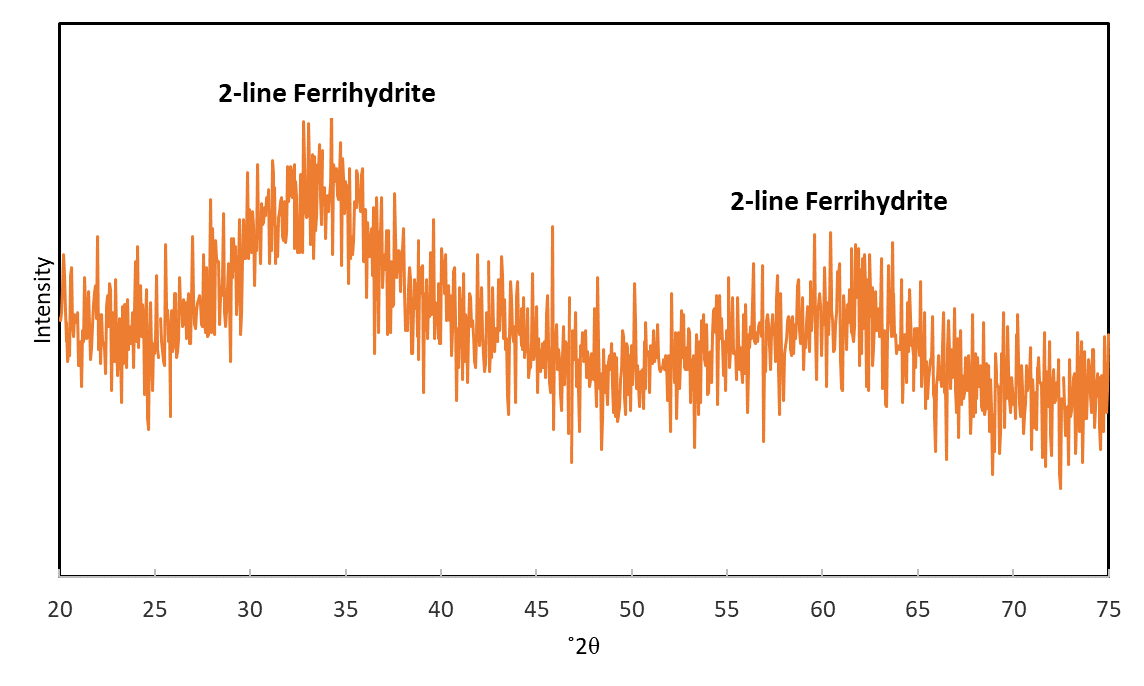


**Supplementary Figure S1.** XRD data for synthesized 2-line ferrihydrite. The two labeled broad peaks are characteristic of 2-line ferrihydrite and confirmed we were using the correct Fe mineral phase.

**Supplementary Table S1.** Aqueous carbon concentrations of DOM stock solution.

| **Total Inorganic C** | **Total C** | **Total Organic Carbon** |
| --- | --- | --- |
| **(mg/L)** | **(mg/L)** | **(mg/L)** |
| 1.58 | 2029.98 | 2028.40 |
|  |  |  |

**Supplementary Table S2.** Aqueous elemental composition of DOM stock solution.

| **Al** | **Ca** | **Cu** | **Fe** | **K** | **Mg** | **Na** | **P** | **S** | **Zn** | **As** | **Pb** |
| --- | --- | --- | --- | --- | --- | --- | --- | --- | --- | --- | --- |
| **(mg/l)** | **(mg/l)** | **(mg/l)** | **(mg/l)** | **(mg/l)** | **(mg/l)** | **(mg/l)** | **(mg/l)** | **(mg/l)** | **(mg/l)** | **(mg/l)** | **(mg/l)** |
| 4.470 | 152.461 | 2.340 | 7.123 | 30.359 | 57.734 | 3.849 | 43.804 | 18.956 | 2.148 | 0.045 | 0.154 |
|  |  |  |  |  |  |  |  |  |  |  |  |
